# Adolescents' social media posting, social support, and the moderating role of tech attitudes and self-esteem: a 2-year longitudinal study

**DOI:** 10.3389/fpsyg.2025.1561581

**Published:** 2025-08-18

**Authors:** Tingting Fan, Lydia Bliss, Angela Calvin, Ellen Selkie

**Affiliations:** University of Wisconsin-Madison, Madison, WI, United States

**Keywords:** social media, social support, self-esteem, technology attitudes, adolescence

## Abstract

**Introduction:**

This study investigates the reciprocal relationship between adolescents' social media posting behaviors and perceived social support over a 2-year period.

**Methods:**

Using a longitudinal design and observational data on posting frequency, we examined whether posting predict perceived social support from close friends and classmates and whether increased social support, in turn, predicts more frequent posting. We also explored the moderating roles of individual differences in self-esteem and attitudes toward technology.

**Results:**

Our findings revealed a reciprocal relationship within close friend networks. More perceived support from close friends in mid adolescence was linked to more posting in late adolescence, which in turn was associated with more support received from participants' close friend networks in late adolescence. This bidirectional association was not observed within classmate networks. Besides, adolescents from low SES backgrounds and those with lower self-esteem demonstrate significant benefits from social media posting, receiving more social support from close friends, while those with negative attitudes toward technology experienced diminished benefits from posting behaviors in receiving social support.

**Discussion:**

This study sheds insight on the relationship between social networks and social media use by highlighting the ways in which social support relate to adolescents' online behaviors, which further advance our knowledge of how adolescent's preexisting personal traits and posting online interact impact social support that adolescents received.

## Introduction

Adolescence is a distinct period marked by rapid physical, psychological, cognitive, and social changes (Steinberg and Morris, 2001). During this transformative time, maintaining positive wellbeing is crucial for adolescents as they navigate these shifts and adapt to emerging challenges with peers. As the most active users of social media, adolescents often turn to these online platforms to seek social support from peers to overcome challenges (Frison and Eggermont, 2015; Toma et al., 2020). This study aims to investigate the reciprocal relationship between adolescents' social media posting and social support from peers over time and to explore the individual differences that influence this process longitudinally.

Social support, broadly defined as assistance provided by others to help individuals manage stress through emotional or material means (Cohen, 2004), is widely recognized as a protective factor during this period. It serves as a buffer against stress, promotes resilience and facilitates adaptive outcomes, such as including higher life satisfaction (Blau et al., 2018; You et al., 2018), enhanced self-esteem (Alshammari et al., 2021; Bum and Jeon, 2016; Kumar et al., 2014), and reduced depressive symptoms (Chang et al., 2018; Cohen and Wills, 1985).

Social support is multifaceted sourced from various networks, including family (Rothon et al., 2012), friends (Rigby, 2000), and classmates (Auerbach et al., 2011). During adolescence, however, the dynamics of social support shift significantly from family-centered to peer-focused, as adolescents spend more time at school and with peers (Collins and Laursen, 2004). Close friendships and relationships with classmates become particularly important. Research shows that high-quality friendships can protect adolescents against depressive symptoms and enhance emotional wellbeing (Chang et al., 2018). As far as classmate support, adolescents often rely on their school-based social networks for emotional and instrumental support (Hombrados-Mendieta et al., 2012). However, negative interactions, such as peer rejection from classmates, could also lower classmate social support (Lev-Wiesel et al., 2006). As a result, the social support adolescents receive from close friends may differ from the experiences they have with classmates. Understanding these distinct sources of support is essential for providing a nuanced view of how peer relationships.

Adolescents from low socioeconomic status (SES) families show even more significant reliance on peer support compared to their mid-to-high SES counterparts. Low-SES adolescents often face greater chronic stress within their family and community environments, making school a vital context for building social capital (Delaruelle et al., 2021). These adolescents receive greater peer support at school compared to their counterparts from higher SES backgrounds (Bermejo-Martins et al., 2023). Despite this distinction, a large portion of the current literature on social support concentrates on middle-class teenagers and focuses on support within close friendships ignoring classmate support, leaving gaps in our knowledge of the distinct experiences of adolescents from lower socioeconomic backgrounds and different kinds of social support. To understand social support among low-SES adolescents, it is essential to look at how social support works in their lives.

### Social media use and its role in adolescent social support

Social media drastically changed how adolescents communicate and seek social support. Adolescents frequently use social media to establish and sustain social relationships, which can improve their perception of social support (Valkenburg and Peter, 2007; Yue et al., 2023). Social media have a positive effect on adolescent wellbeing by building resilience, particularly in the face of adversity like the COVID-19 pandemic (Chen et al., 2022; Yue et al., 2023).

Yet the literature is far from uniform. Some studies find positive associations between social media use and support (Rains et al., 2015; Steinfield et al., 2009), whereas other studies find null, or even negative relationships (Blahošová et al., 2024; Hall, 2018; Utz and Breuer, 2017). These conflicting findings may stem from inconsistencies in how social media use and social support are measured and defined across studies (Meng et al., 2016). For instance, some studies differentiate between active and passive media use, while others only consider general frequency of use (Verduyn et al., 2017). To clarify our approach, the present study specifically focuses on adolescents' posting—a form of active social media use. Compare to private messaging, such as on Instagram or Facebook messenger or Snapchat, posting normally reaches a broader audience attracting wider attention. It can involve creating original content or resharing others' content, allowing adolescents to curate their online self-presentation and manage how they are perceived, potentially shaping their self-image. Frequent posting is linked to greater audience interaction, which may enhance adolescents perceived social support by generating likes, comments, and shares that boost their social media presence (Danielsen et al., 2024).

### The need to explore reciprocal relationship between active social media use and social support

Uses and gratifications theory (Wu et al., 2010) posits that adolescents actively choose specific social media behaviors to fulfill underlying needs. For instance, adolescents may post online to connect with peers, build social identity, seek validation, and receive social support. These online interactions help satisfy social needs and can enhance adolescents' wellbeing, suggesting a directional influence from social media posting to social support. However, (Meng et al. 2016) highlighted that a unidirectional perspective—where social media use drives social support—may fail to account for the possibility that social support can also influence social media engagement. According to social support theory (House, 1981), individuals who feel supported by others are more motivated to engage in social behaviors. For instance, when adolescents feel supported by others, they are more likely to engage in sustained and intimate interactions with them. Adolescents with preexisting social support may be more likely to maintain communication with peers after school on social media compared to adolescents who do not have high social support. Previous literature found that supportive climate provided by peers would encourage self-disclosure to others (Lin et al., 2021). Therefore, high perceived social support may encourage adolescents to share content and express themselves online, confident in the likelihood of positive feedback and reinforcement from their peers. This supportive dynamic can increase the frequency of posting, as adolescents anticipate validation and a strengthening of their social bonds, regardless of their physical location. However, most existing research relies on cross-sectional design, which are limited in capturing reciprocal influences over time. (Meng et al. 2016) advocated for longitudinal studies to better understand how social support and social media use influence each other over time. Recently, a few studies have begun addressing this gap. For example, (Blahošová et al. 2024) used ecological momentary assessment to test whether adolescents' social media use and social support from the previous day predicted their perceived social support and media use the following day. This study found no significant effects at either the between- or within-person levels. Similarly, (Yue et al. 2023) investigated this relationship longitudinally comparing pre and post pandemic differences, finding an indirect association between social media use and social support, mediated by perceptions of online network responsiveness. These recent findings highlight the complexity of the relationship between perceived social support and media use. Given the conflicting findings surrounding the significance, direction, and reciprocal nature of the relationship between social media use and perceived social support, the present study aims to further investigate this connection using a longitudinal design to provide a comprehensive understanding of the temporal dynamics over time.

### The role of moderating factors

While previous research has examined the associations between social media use and perceived social support, findings suggest that perceived social support alone may not fully account for the link between social media use and social support (Yue et al., 2023). Rather, this relationship may depend on certain moderating factors, such as individual differences that influence the strength or direction of the association. Exploring moderating factors will help provide explanations as to why social media use and social support may be sometimes related and other times not.

Self-esteem is one moderating factor that may explain how adolescents perceive and respond to social support on social media and off as well as their posting. Adolescents with high self-esteem are more likely to experience increased perceived social support through being more resilient to negative evaluation and are less likely to engage in negative online comparison (Valkenburg et al., 2017). Conversely, adolescents with low self-esteem may be more vulnerable to social comparisons and may interpret a lack of engagement, such as a lack of likes or comments, as social rejection, which could negatively affect their sense of support and wellbeing (Forest and Wood, 2012). Therefore, compared to adolescents with lower self-esteem, individuals with greater self-esteem may be more likely to post online. It remains unclear, though, how self-esteem influences the connection between social media posting and perceived social support.

Adolescents' attitudes toward technology also play a crucial role in shaping their social media use. Previous research has shown that attitudes toward technology are significantly associated with the amount of time individuals spend on social media activities. Specifically, a positive attitude toward technology is positively correlated with daily social media use, including time spent online and on social networking sites. Conversely, a negative attitude toward technology is associated with reduced engagement in daily media activities, such as chatting and emailing (Rosen et al., 2013). Adolescents with positive attitude toward technology are more likely to communicate with others online, which can help them maintain social support from offline relationships while potentially fostering support from an online community. However, the benefits of social media on perceived support may be diminished for those with ambivalent or negative attitudes, as they are less likely to view online interactions as beneficial or meaningful (Twenge et al., 2018). Adolescents with negative perceptions of technology may miss opportunities to stay consistently connected with their online and offline social networks, leading to fewer interactions and limiting their ability to receive ongoing support.

## The present study

The present study addresses two primary research objectives to advance our understanding of the interplay between adolescents' social media posting behaviors and perceived social support. First, we aim to investigate the reciprocal relationship between adolescents' posting behaviors and their perceived social support over a 2-year period. This longitudinal approach allows us to explore whether increased social support predicts more frequent posting behaviors, and whether posting behaviors in turn contribute to perceived social support. Through this design, we aim to provide a more nuanced understanding of the complex, relationship between social media use and social support during adolescence. By focusing exclusively on posting, we emphasize how this specific type of social media use may help adolescents build and sustain peer support. Posting allows adolescents to curate their online self-image and fosters audience engagement (e.g., likes and comments) that can enhance their perceived social support (Pang, 2020). Additionally, the level of support adolescents perceive from peers may, in turn, influence their posting frequency, as they seek or respond to social attention and validation. Unlike prior studies that have often relied on self-reported data, which can be limited by recall bias and social desirability, this study adopts an observational approach, directly recording adolescents' posts on social media platforms. This objective measure offers more robust data on posting frequency, allowing for a “real world,” direct examination of how online interactions relate to perceived social support.

The second objective is to explore how individual differences—specifically self-esteem and attitudes toward technology—moderate the relationship between posting behavior and social support. Examining these moderating factors allows us to identify individual characteristics and attitudes under which active social media use may either bolster or limit perceived support. We hypothesize that adolescents with higher self-esteem will experience a stronger positive relationship between social media posting behavior and perceived social support. Additionally, adolescents who hold negative attitudes toward technology may experience a weakened or even negative relationship between posting behavior and perceived social support, as these attitudes is associated with decreased online interactions with others (Rosen et al., 2013). In addressing these objectives, this study provides a nuanced, reciprocal examination of the relationship between social media use and social support, as well as adds to the present literature identifying about how individual differences impact this relationship.

## Method

### Participants & procedure

Participants for the present study were drawn from a longitudinal cohort originally designed to investigate maternal feeding practices, which was recruited between 2009 and 2011 (Lumeng et al., 2014; Miller et al., 2013) when children were enrolled in Head Start, a preschool program aimed at serving low-income families. Thus, all participants in both the ABC study and the current study came from low socioeconomic status (SES) backgrounds. For more detailed participant information from the ABC study, refer to Calvin et al. (2025).

During recruitment for the current study, both participants and their parents were informed that data collection would occur five time points across a 2-year period. The study involved not only self-reported surveys but also a specially designed protocol for observing participants' social media activity by following them or friending them on their social media accounts (see the Measures section for further details). The observations focused on Facebook, Twitter, and Instagram, which were selected based on their popularity among adolescents at the time (Anderson and Jiang, 2018), as well as the feasibility of tracking and recording posts on these platforms. Only participants who had at least one active account on these platforms were eligible to participate.

After obtaining electronic informed consent from parents and electronic assent from adolescents, 152 participants agreed to participate in this follow-up study. After reviewing the data for missing responses, only participants who had completed at least 75% of the surveys were included in the final analysis. This resulted in a sample of 142 participants (93.4% of those consented), with 52.11% identifying as female and 59.90% identifying as White at T1.

Although data were collected at five time points across the 2-year period across participants' middle school and high school transition period, measures of social support were only collected at the first (T1) and final (T2) time points. As a result, the present analysis focuses on data from these two waves. At T1, the mean age of participants was 13.29 years (SD = 0.53), and at T2, their mean age was 15.33 years (SD = 0.55). Power analysis using G^*^Power version 3.1 Faul et al., 2009 indicated that a sample size of 98 would provide sufficient statistical power (≥0.80) at an alpha level of 0.05 and an effect size of *f*^2^ = 0.15 (cf. Mayr et al., 2016). The study was approved by the authors' university's Institutional Review Board (ID: 2021-0848).

For further details on the study background, recruitment process, and participant demographics, refer to Calvin et al. (2025).

### Measures

The present study employed two primary data collection methods: social media observations and self-reported surveys. Social media observations were used to gather data on participants' number of posts, while self-reported surveys captured participants perceived social support from friends and classmates and individual differences, including self-esteem and attitudes toward technology use. All measures were collected at two time points, T1 and T2, spaced 2 years apart.

#### Numbers of social media posts

To minimize inaccuracies from self-reported recall of social media posting activity, this study employed a direct observation method. Participants granted the research team permission to “friend” or follow their accounts on Facebook, Instagram, and Twitter. This allowed researchers to passively monitor participants' social media activity without influencing their behavior. At both T1 and T2 (the start and end of the 2-year period), the research team accessed participants' accounts on these platforms and recorded the total number of posts made in a 1-month period.

#### Perceived social support

Perceived social support from friends and classmates was measured using subscales from the Child and Adolescent Social Support Scale (CASSS; Malecki et al., 2000). Social support from classmates was assessed using a 12-item subscale (e.g., “My classmates like most of my ideas and opinions”) with responses rated on a six-point Likert scale ranging from 1 (Never) to 6 (Always) (α = 0.95). Similarly, perceived social support from a close friend was measured using another 12-item subscale (e.g., “My close friend helps me when I need it”), with identical Likert scale (α = 0.95). Higher scores indicate higher social support.

#### Self-esteem

Participants' self-esteem was assessed using 10 items from the Rosenberg Self-Esteem Scale (Rosenberg, 1965), which included items such as, “I feel that I am a person of worth, at least on an equal plane with others.” Participants rated their agreement with each statement on a four-point Likert scale, ranging from 1 (Strongly Disagree) to 4 (Strongly Agree). Higher scores indicated higher self-esteem (α = 0.87).

#### Attitude toward technology

Negative attitudes toward technology were measured using three items from the Media Usage Subscale of the Media and Technology Usage and Attitudes Scale (Rosen et al., 2013). An example item is “New technology makes people more isolated.” Participants rated their agreement on a five-point Likert scale, ranging from 1 (Strongly Disagree) to 5 (Strongly Agree). Higher scores reflected a more negative attitude toward technology (α = 0.78).

#### Covariates

Participants reported their sex and race, which were included as covariates in the analysis.

## Results

Data analysis was conducted using R, with all numeric variables centered to their mean values to ensure the validity of the results. Descriptive statistics and correlations regarding the number of media posts, perceived social support at Time 1 (T1) and Time 2 (T2), as well as other key variables in the present study, are shown in Table 1.

**Table 1:** Correlation between all key variables.

**Variables**	**1**	**2**	**3**	**4**	**5**	**6**	**7**	**8**	**M(SD)**
1. Posting at T1	–	0.21^*^	−0.10	−0.10	−0.04	0.06	−0.06	−0.02	8.63 (24.10)
2. Posting at T2	0.21^*^	–	−0.08	0.13	−0.12	0.19^*^	−0.16	−0.16	6.75 (23.20)
3. Classmates social support at T1	−0.10	−0.08	–	0.55^***^	0.50^***^	0.26^*^	0.10	0.09	3.60 (1.11)
4. Close friends social support at T1	−0.10	0.13	0.55^***^	–	0.38^***^	0.34^***^	0.12	−0.07	4.41 (1.03)
5. Classmates social support at T2	−0.04	−0.12	0.50^***^	0.38^***^	–	0.51^***^	0.25^**^	0.12	3.54 (1.03)
6. Close friends social support at T2	0.06	0.19^*^	0.26^**^	0.34^***^	0.51^***^	–	0.17^*^	−0.08	4.36 (1.02)
7. Self-esteem at T2	−0.06	−0.16	0.10	0.12	0.25	0.17^*^	–	0.08	2.69 (0.21)
8. Negative attitude toward technology at T2	−0.02	−0.16	0.09	−0.07	0.12	−0.08	0.08	–	3.18 (0.71)

^***^p < 0.001, ^**^p < 0.01, ^*^p < 0.05.

### Reciprocal relationship between media posting and social support

A series of multivariate linear regression analyses were conducted to examine the reciprocal relationship between social media posting and social support. First, we tested whether the number of social media posts at T1 predicted perceived social support from close friends and classmates at both T1 and T2. Next, we tested the reverse paths, examining whether social support from friends and classmates at T1 predicted the number of media posts at both T1 and T2. Finally, we explored whether the number of media posts at T2 predicted social support from close friends and classmates at T2, as well as the reverse relationships. In all models, baseline measures (e.g., social media posting at T1) and covariates such as sex and ethnicity were controlled to ensure the robustness of the findings. Complete results for all multivariate linear regression models are available in the Appendix Tables A2–A4.

The results of the regression model showed that the number of social media posts at T1 did not predict perceived social support from either close friends or classmates at both T1 and T2. Similarly, social support from friends and classmates at T1 did not predict the number of media posts at T1. However, social support from close friends at T1 significantly predicted an increase in the number of social media posts at T2 (β = 0.27, *p* < 0.05).

**Figure 1:**
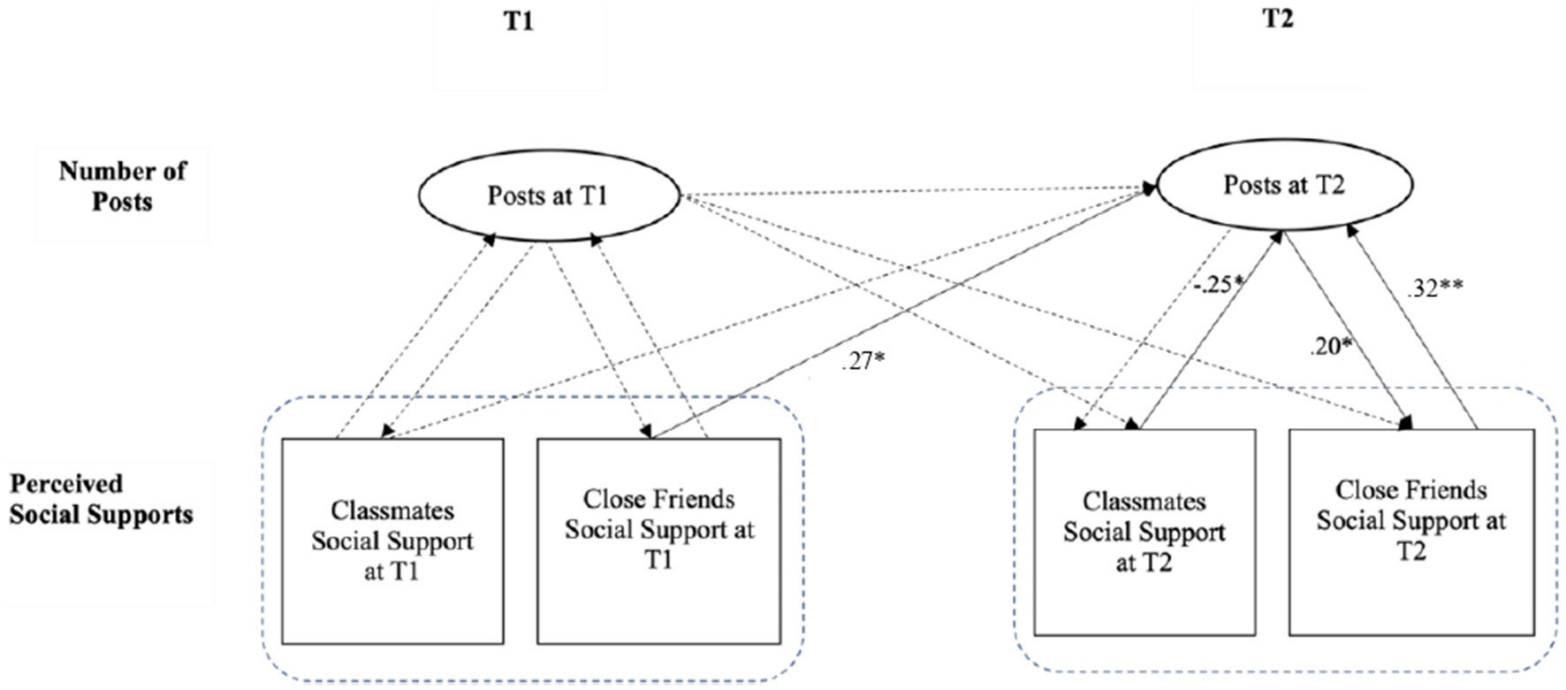
Reciprocal explorations between number of posts and social support. **p < 0.01, *p < 0.05. Solid lines represent significant paths.

The number of social media posts at T2 did significantly predict perceived social support from close friends at the same time point (T2) (β = 0.20, *p* < 0.05). Interestingly, the reverse was also observed: higher social support from close friends at T2 predicted an increase in the number of social media posts at T2 (β = 0.32, *p* < 0.01). However, contrary to expectations, social support from classmates at T2 was associated with a decrease in the number of social media posts at T2 (β = −0.25, *p* < 0.05). Figure 1 presents the integrated results from these models.

The results from the multivariate models reveal a significant reciprocal relationship between social support from close friends and social media posting. Specifically, social support from close friends at T1 predicted an increased number of media posts at T2. In turn, a higher number of posts at T2 was associated with greater perceived social support from close friends at the same time point. This suggests that social media posting does not directly enhance adolescents' perceived social support; rather, it is when adolescents already have supportive friendships that they are more likely to engage in social media posting, which then reinforces the support they receive.

Conversely, the data did not support a similar relationship with classmates. Social support from classmates did not predict an increase in the number of posts over time. In fact, social support from classmates at T1 was associated with a decrease in the number of media posts at T2, indicating that support from classmates may have a different or even opposing effect on social media posting behavior compared to support from close friends.

Figure 1 presents the reciprocal models between social media posting and social support from both close friends and classmates. These findings highlight the distinct roles that different social relationships play in shaping media behaviors. Close friendships appear to foster a positive feedback loop, where increased social media posting strengthens social support, while support from classmates may discourage such behavior.

### Impact of individual differences as moderators

In addition to testing the reciprocal relationship between social media posting and social support, we conducted moderation analyses to examine how individual differences—specifically self-esteem and negative attitudes toward technology—alter this relationship. Given that the reciprocal pathways between the number of posts and social support from close friends and classmates were significant at T2, the moderation analysis focused exclusively on T2 data, using self-reported measures of self-esteem and negative attitudes toward technology at that time. Each moderator was analyzed separately.

To examine whether adolescents' self-esteem and negative attitude toward technology moderate the relationship between perceived social support and social media posting at T2, moderation analyses were conducted for social support from close friends, and from classmates separately. Significant moderation were only observed for close friends support.

#### Impact of self-esteem on the relationship between social support and social media posting

**Figure 2:**
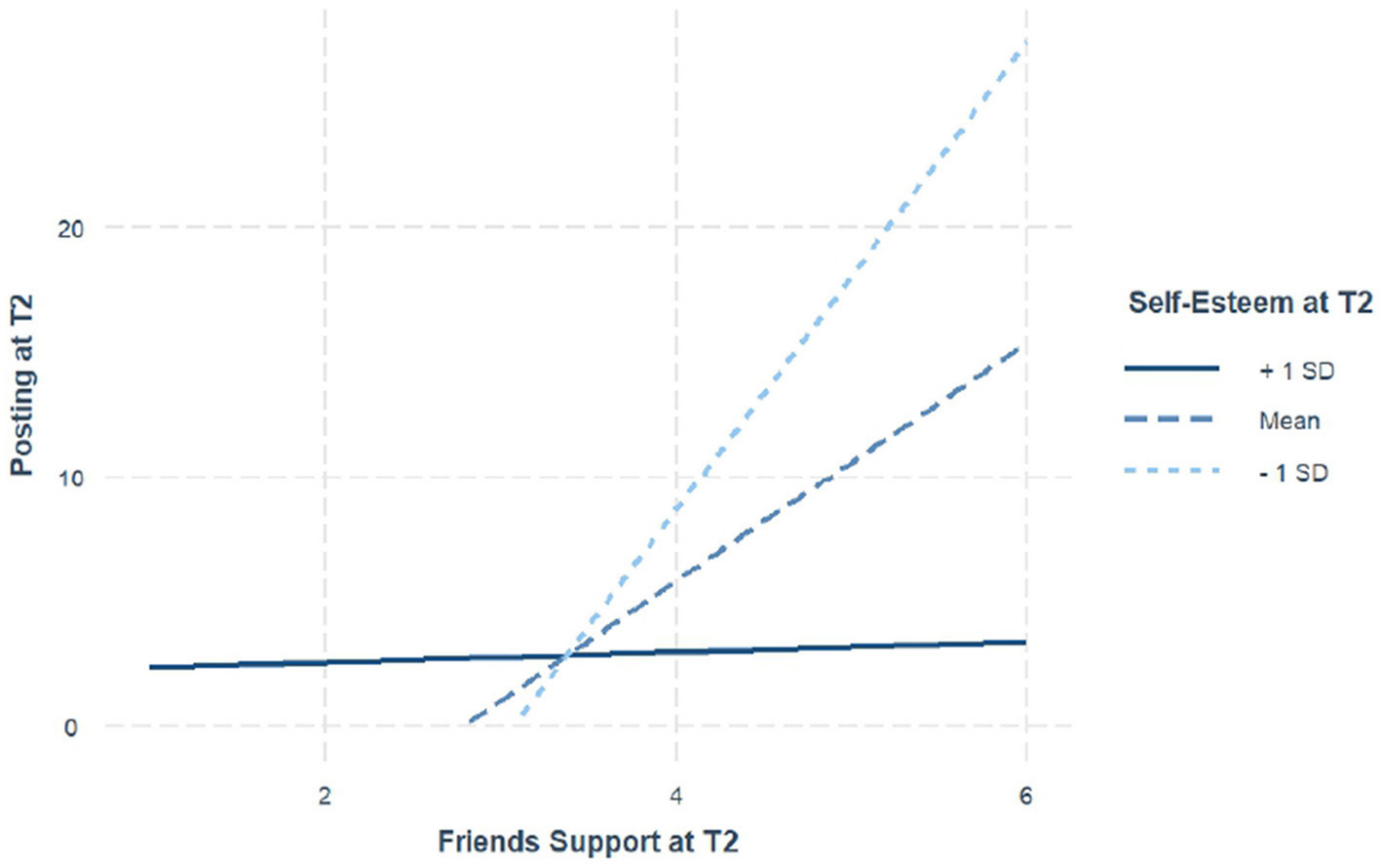
Moderation effect of self-esteem on number of posts and social support from a close friend. Posting at T2 represents total number of posts made at T2. Friends support represents close friend support at T2, which ranges from 1 to 6.

For the moderation analysis involving social support from close friends, significant effects were observed within the overall moderation model that self-esteem moderated the relationship between the pathway from social support to social media posting at T2 [*F*_(5, 136)_ = 5.29, *p* < 0.001]. Most importantly, a significant interaction was found between social support from friends and self-esteem (β = −0.20, *SE* = 0.07, *t* = −2.83, *p* < 0.01), indicating that the positive relationship between social support from friends and social media posting was weaker among adolescents with higher self-esteem. This interaction suggests that while social support from friends generally increases posting behavior, adolescents with higher self-esteem may rely less on external support for their posting activity. Whereas, adolescents with lower self-esteem rely more on their close friends support, which they may receive via posting online. The visualization of this moderation effect is shown in Figure 2.

#### Impact of negative attitudes on the relationship between social support and media posting

**Figure 3:**
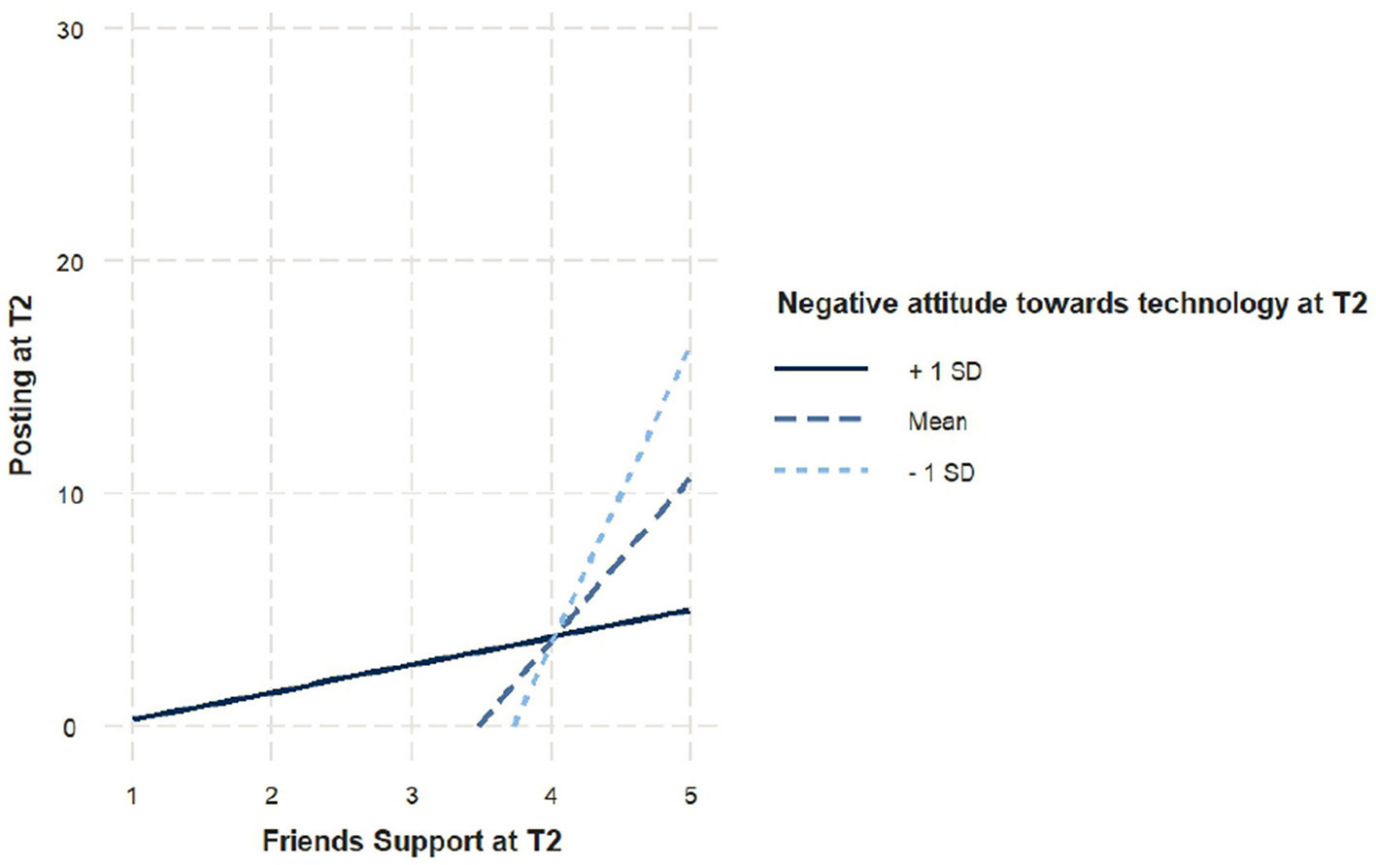
Moderation effect of negative attitude toward technology on number of posts and social support from a close friend. Posting at T2 represents total number of posts made at T2. Friends support represents close friend support at T2, which ranges from 1 to 6.

Negative attitudes toward technology moderate the same pathway in the same direction, the overall model was significant [*F*_(5, 136)_ = 5.28, *p* < 0.001]. As expected, the interaction between social support and negative attitudes was also significant (β = −0.25, *SE* = 0.07, *t* = −3.48, *p* < 0.001). This suggests that adolescents with higher negative attitudes toward technology exhibited a weaker positive relationship between social support from close friends and their number of media posts at T2. This suggests that for individuals with more negative attitudes toward technology, the social support they receive from close friends is less effective in encouraging social media posting, potentially limiting their ability to benefit from further social support in the future. The visualization of this moderation effect was shown in Figure 3.

## Discussion

### Reciprocal relationship between social media posting and social support

This study aimed to examine the reciprocal relationships between social media posting and perceived social support from two peer networks—close friends and classmates—over a 2-year period using a two-wave longitudinal design.

#### Social media posting and support from close friends

The findings indicated that while no significant association was observed between social media posting and social support concurrently, earlier in adolescence, support from close friends at T1 predicted an increased frequency of media posting at T2. In turn, more frequent media posting at T2 was associated with greater perceived support from close friends at T2. This mutually reinforcing relationship suggests that adolescents who receive substantial support from close friends may be encouraged to engage more on social media, which can further enhance their perception of social support within close friendships. This feedback loop appears to be initiated by existing social support from close friends at a younger age or a history of social support over 2 years, rather than by an adolescent's media posting behavior, aligning with social support theories (House, 1981), in other words, that adolescents who feel secure and supported are more likely to use social media as a means to strengthen and maintain their social bonds. This feedback loop further indicated that supportive online environment reinforces the strength of close friendships. However, it is noteworthy that this feedback loop did not originate from adolescents' posting behavior alone, indicating that posting is not necessarily an effective strategy to obtain social support. This finding contrasts with previous studies that emphasize active social media use as a means of increasing social support (e.g., Carpenter et al., 2018). Rather, adolescents' posts may primarily serve self-presentation, self-expression, and validation-seeking purposes, rather than to elicit tangible support from friends. Furthermore, broadcasting content to a broad audience, as opposed to engaging in direct social interactions like private messaging, may not foster meaningful connections. Prior research has indicated that meaningful social interactions are more likely to occur through one-on-one conversations (e.g., private messaging) rather than through public posts to a large audience (Carpenter et al., 2018).

#### Social media posting and support from classmates

In contrast to the reciprocal relationship observed with close friends, higher perceived social support from classmates at T1(in middle adolescence) as associated with a decrease in media posting by T2 (in late adolescence). This unexpected finding suggests that support from classmates may not encourage self-presentation on social media in the same manner as support from close friends. Given that this study did not assess the content of adolescents' posts, it is possible that adolescents selectively shared content aimed at strengthening close friendships, while the same content may have been less relevant or appealing to classmates. Additionally, classmates' support could reflect implicit social norms that discourage excessive posting or promote offline interactions over online engagements, thereby reducing the need for frequent online self-presentation. Alternatively, support from classmates may sufficiently fulfill adolescents' social needs within the school environment itself, diminishing the perceived need for additional online engagement.

Additionally, the unique circumstances of data collection, which spanned the beginning and end of the COVID-19 lockdown, may have influenced these findings. In the initial year of data collection, most participants had not yet experienced lockdown, whereas in the second year, social media posting may have become a primary means of communication with peers due to social restrictions (Cauberghe et al., 2021). Besides, adolescents who participated in the present study might experiencing school transitions from middle to high school, where they might experience unstable classmates during this transition. As a result, participants in our study might have little chance to establish new and stable relationships with their classmates face-to-face. Thus, they might have unstable classmates as potential audience in the online context. This might explain why classmates' bonds play a different role compared to the close friend bonds. These contextual factors could have heightened the role of social media in maintaining close friendships, thereby reinforcing the observed feedback loop.

### Individual differences as moderators

In the current study, the role of individual differences—specifically self-esteem and negative attitudes toward technology—emerged as critical in moderating the relationship between social support and media posting among adolescents.

#### Self-esteem as a moderator

Self-esteem plays a role in moderating the relationship between social support from close friends and media posting behavior. Adolescents with higher self-esteem demonstrated a weaker link between perceived social support and media posting at Time 2 (T2). This aligns with previous studies, which suggest that individual traits shape social media use and its impact on social support outcomes (Chen et al., 2022). The results in the present study indicates that adolescents with high self-esteem do not necessarily seek social support or validation through media posts. It might because of that these adolescents may fulfill their needs through internal sources rather than seeking external validation via social media. Conversely, those with lower self-esteem may rely more on social media posting to receive support from close friends, helping to meet their needs for validation and social affirmation. Consequently, adolescents with lower self-esteem may engage more heavily in media posting to gain emotional and tangible resources.

#### Negative attitudes toward technology as a moderator

Consistent with our expectations, the findings indicate that negative attitudes toward technology significantly moderate the relationship between social support from close friends and media posting. Adolescents who view technology with skepticism—lacking trust and perceiving potential harm to youth—are less likely to benefit from media posting. Although media posting can provide social support from close connections, holding negative views about technology may hinder adolescents from engaging actively online with friends, limiting their ability to receive continued support through digital channels. This finding underscores the complex role of technology in adolescents' peer relationships; those who favor in-person interaction over digital engagement may find it challenging to derive social benefits, such as support from friends, through online platforms. These results highlight the importance of considering individual attitudes toward technology when examining adolescents' digital behaviors and associated outcomes.

In general, these results highlight the critical role of individual differences in shaping the dynamics of social support and media posting. While social support from close friends generally promotes social media posting, this effect can be amplified or diminished depending on an adolescent's level of self-esteem and attitude toward technology.

## Contributions

The present study makes three key contributions. First, it highlights that social media posting does not uniformly yield social support across all peer networks. Rather, the impact of social media posting on perceived social support appears contingent on the type and quality of offline relationships. The divergent effects observed between close friends and classmates underscore the importance of considering how different social ties shape online behaviors. Prior research has demonstrated that individuals are more likely to offer supportive engagement, such as comments, when they feel a close relationship with the poster, suggesting that relationship closeness enhances the likelihood of supportive online interactions (Rousseau et al., 2019).

Second, by focusing on adolescents from low-SES backgrounds, this study emphasizes the distinct challenges they may face in navigating peer relationships and social media use. These adolescents may perceive lower acceptance from broader social circles, particularly classmates, and feel less comfortable sharing personal challenges or emotions openly with them. Exposure to negative life events may lead to posts reflecting these experiences, which classmates may find less relatable or engaging. Consequently, these adolescents may prioritize sharing within close friend networks, where they anticipate understanding and empathy, over engaging with classmates, from whom they may expect less supportive feedback. Thirdly, the present research enrich the understanding about how individual's personal heterogeneity may impact the relationship between social media posting and social support. This dynamic adds complexity to the relationship between social media posting and social support and underscores the importance of considering social networks and individual differences when examining adolescents' online behaviors. These findings collectively underscore the multifaceted nature of adolescent social media engagement and point to the need for future research that takes into account the influence of network differences, socioeconomic background, and individual differences.

## Limitations & future directions

First, there are additional aspects of social media use not considered in the present study. Notably, the content and valence of participants' posts was not measured, leaving it unclear whether posts reflected everyday activities or more emotionally charged topics (e.g., achievements vs. challenges or failures). Positive content is generally more likely to elicit social support from a broader audience than negative content. Second, adolescents' posting behavior can differ based on their motivations (Jarman et al., 2021), which were also not examined. Some individuals may post simply to document their daily lives or manage self-image rather than to seek social support. Third, our observations depended on “friending” participants and viewing only the content they allowed our researcher accounts to see. Adolescents could restrict post visibility (e.g., limit certain posts to “close friends”) or rely on private channels such as direct messaging to communicate, leaving portions of their active social media use unobserved and potentially attenuating associations with perceived support. Future research should incorporate these varied aspects of media use as well as integrate multi-method data sources (e.g., self-report survey, objective observations) to capture a full ecology of adolescents' online activities and more precisely map the relationship between media use and social outcomes.

Additionally, the sample size in the present study was relatively limited. While we calculated effect sizes to ensure appropriate analysis, the small sample size and data structure restricted our ability to conduct more complex analyses, such as those examining reciprocal relationships or establishing causal links. Furthermore, this study specifically sampled adolescents from low-SES backgrounds. Future research should examine reciprocal relationships and individual differences using a larger sample, including adolescents from all SES backgrounds, to enhance generalizability.

## Conclusion

This study highlights the reciprocal relationship between adolescents' social media posting and perceived social support, particularly within different social networks. Social support from close friends fosters a feedback loop of increased posting and enhanced support, whereas support from classmates shows a contrasting pattern of reduced posting. These findings underscore the role of relationship quality in shaping online behaviors. Moderating effects of self-esteem and attitudes toward technology further illustrate how individual traits influence adolescents' ability to derive social benefits from posting. Importantly, the study contributes to future intervention strategies by highlighting the need to leverage close peer networks and address individual differences to enhance social support for low-SES adolescents, thereby promoting their wellbeing. Future research should explore these dynamics across diverse contexts and consider other forms of social media engagement to deepen understanding of adolescent social development.

## Data Availability

The datasets presented in this article are not readily available because of ethical and privacy considerations. Requests to access the datasets should be directed to Tingting Fan.
