# Supplementary material for: Adolescents' social media posting, social support, and the moderating role of tech attitudes and self-esteem: a 2-year longitudinal study
Source: Front Psychol. 2025 Aug 18;16:1561581. doi: 10.3389/fpsyg.2025.1561581 (PMC12399651; doi:10.3389/fpsyg.2025.1561581)
Supplement: Supplementary file 1 [file Data_Sheet_1.pdf]

APPENDIX A

Table 2a.

Multivariate Linear Regression Model Using Perceived Social Support at T1 Predicting Number of Posts at T1

	B(SE)	t	p
Intercept	0.01(0.09)	0.07	.95
Friend social support at T1	-0.07(0.11)	-0.68	.50
Classmates social support at T1	-0.05(0.11)	-0.44	.66
Covariates			
White (1=Yes)	4.41(1.03)	0.54	.59
Female(1=Yes)	3.54(1.03)	-0.11	.91
R^2 / adj. R^2	.015/-.014		
F(df)	0.52(4,134)		.720

Table 2b.

Multivariate Linear Regression Models Using Number of Posts at T1 Predicting Perceived Social Support at T1

	Model for Classmates Social Support at T1			Model for Friend Social Support at T1		
	B(SE)	t	p	B(SE)	t	p
Intercept	0.01 (0.08)	0.07	.95	0.01 (0.08)	0.15	.883
Number of Posts at T1	-0.08 (0.08)	-1.00	.32	-0.09 (0.08)	-1.07	.286
Covariates						
White (1=Yes)	-0.24 (0.08)	-2.90	.004**	-0.11 (0.08)	-1.34	.182
Female(1=Yes)	-0.14 (0.08)	-1.64	.103	0.08 (0.08)	1.01	.316
R^2 / adj. R^2	.084/.063			.030 / .009		
F(df)	4.11(3,135)		.008**	1.41 (3, 136)		.241

Note. **p < 0.01.

Table 3a.

Multivariate Linear Regression Model Using Social Support at T2 Predicting Number of Posts at T2

	B(SE)	t	p
Intercept	0.00(0.09)	-0.04	.970
Friend social support at T2	0.32(0.11)	2.89	.005**
Classmates social support at T2	-0.25(0.11)	-2.15	.034*
Covariates			
White (1=Yes)	0.09(0.10)	0.94	.348
Female(1=Yes)	0.12(0.10)	1.25	.214
R^2 / adj. R^2	.122/.088		
F(df)	3.61(4,104)		.008**

Note. **$p < 0.01$, *$p < 0.05$.

Table 3b.

Multivariate Linear Regression Models Using Number of Posts at T2 Predicting Perceived Social Support at T2

	Model for Classmates Social Support at T2			Model for Friend Social Support at T2		
	B(SE)	t	p	B(SE)	t	p
Intercept	0.02 (0.09)	0.25	.805	0.01 (0.10)	0.06	.956
Number of Posts at T2	-0.07 (0.09)	-0.70	.485	0.20(0.10)	2.04	.044*
Covariates						
White (1=Yes)	-0.14 (0.10)	-1.52	.132	0.03 (0.10)	0.27	.791
Female(1=Yes)	-0.24 (0.10)	-2.51	.014*	-0.07 (0.10)	-0.68	.499
R^2 / adj. R^2	.086/.060			.042 / .015		
F(df)	3.28(3,105)		.024*	1.41 (3, 105)		.210

Note. *p < 0.05.

Table 4a.

Multivariate Linear Regression Model Using Social Support at T1 Predicting Posting number at T2

	B(SE)	t	p
Intercept	0.00(0.09)	-0.09	.928
Friend social support at T1	0.27(0.12)	2.30	.023*
Classmates social support at T1	-0.19(0.11)	-1.64	.104
Covariates			
White (1=Yes)	0.12(0.09)	1.33	.187
Female(1=Yes)	0.13(0.09)	1.38	.170
R^2 / adj. R^2	.095/.061		
F(df)	2.83(4,108)		.028*

Note. *$p < 0.05$.

Table 4b.

Multivariate Linear Regression Models Using Posting Number at T1 Predicting Perceived Social Support T2

	Model for Classmates Social Support at T2			Model for Friend Social Support at T2		
	B(SE)	t	p	B(SE)	t	p
Intercept	0.04 (0.09)	0.41	.686	0.00 (0.10)	0.03	.975
Posting at T2	-0.12 (0.11)	-0.11	.911	0.11(0.12)	0.90	.371
Covariates						
White (1=Yes)	-0.15 (0.09)	-1.64	.105	0.03 (0.10)	0.33	.745
Female(1=Yes)	-0.27 (0.09)	-2.92	.004*	-0.04 (0.10)	-0.38	.708
R^2 / adj. R^2	.091/.068			.009 / -.018		
F(df)	3.7(3,108)		.014*	0.34 (3, 108)		.795

Note. *p < 0.05.
